# Supplementary material for: Modeling glioblastoma heterogeneity as a dynamic network of cell states
Source: Mol Syst Biol. 2021 Sep 16;17(9):e10105. doi: 10.15252/msb.202010105 (PMC8444284; doi:10.15252/msb.202010105)
Supplement: Supplementary file 6 — Source Data for Figure 5 [file MSB-17-e10105-s004.zip › Figure5A_sourcedata/GSEA_3017/nosigdb_stateB.GseaPreranked.1622795021725/NB_KI_CLUSTER2.html]

Details for gene set NB\_KI\_CLUSTER2[GSEA]

|  || Dataset | state43017 |
| Phenotype | NoPhenotypeAvailable |
| Upregulated in class | na\_neg |
| GeneSet | NB\_KI\_CLUSTER2 |
| Enrichment Score (ES) | -0.41765067 |
| Normalized Enrichment Score (NES) | -1.9644446 |
| Nominal p-value | 0.015544041 |
| FDR q-value | 0.0127629405 |
| FWER p-Value | 0.049 |
Table: GSEA Results Summary

  

Fig 1: Enrichment plot: NB\_KI\_CLUSTER2      
 Profile of the Running ES Score & Positions of GeneSet Members on the Rank Ordered List

  

| PROBE | GENE SYMBOL | GENE\_TITLE | RANK IN GENE LIST | RANK METRIC SCORE | RUNNING ES | CORE ENRICHMENT || 1 | CXXC5 |  |  | 210 | 0.408 | -0.1974 | No |
| 2 | EFNB2 |  |  | 374 | 0.317 | -0.3504 | No |
| 3 | AHI1 |  |  | 425 | 0.299 | -0.3555 | Yes |
| 4 | PPP1R9A |  |  | 435 | 0.295 | -0.3062 | Yes |
| 5 | PHIP |  |  | 440 | 0.295 | -0.2503 | Yes |
| 6 | MEST |  |  | 441 | 0.294 | -0.1891 | Yes |
| 7 | TTLL7 |  |  | 497 | 0.281 | -0.2044 | Yes |
| 8 | CPNE2 |  |  | 535 | 0.275 | -0.1970 | Yes |
| 9 | PBX1 |  |  | 567 | 0.270 | -0.1825 | Yes |
| 10 | ICA1L |  |  | 621 | 0.259 | -0.1999 | Yes |
| 11 | PRKACB |  |  | 636 | 0.257 | -0.1652 | Yes |
| 12 | MCC |  |  | 664 | 0.253 | -0.1488 | Yes |
| 13 | DSEL |  |  | 709 | -0.297 | -0.1463 | Yes |
| 14 | EPN2 |  |  | 711 | -0.305 | -0.0841 | Yes |
| 15 | ZEB1 |  |  | 717 | -0.317 | -0.0248 | Yes |
| 16 | MIR4458HG |  |  | 732 | -0.384 | 0.0363 | Yes |
Table: GSEA details [plain text format]

  

Fig 2: NB\_KI\_CLUSTER2: Random ES distribution      
 Gene set null distribution of ES for **NB\_KI\_CLUSTER2**
